# Microbial Musings – March 2020

**DOI:** 10.1099/mic.0.000914

**Published:** 2020-04-01

**Authors:** Gavin H. Thomas

**Affiliations:** ^1^​ Department of Biology, University of York, PO Box 373, York YO10 5YW, UK

**Keywords:** editorial, microbiology

Microbiology is still at the forefront of everybody's minds during March as Covid-19 continues to spread and is now officially designated as a pandemic by the World Health Organization (WHO). Some of our members are taking important roles in engaging with the media, including *Microbiology* Editor Willem van Schaik (@WvSchaik) along with *Microbial Genomics* Editor Alan McNally (@alanmcn1) and *Journal of General Virology* Editor-in-Chief Paul Duprex (@10queues), amongst others. To reduce the rate of spread and keep my students and staff healthy, we have shut all the research labs at York, UK, in common with other labs around the world. Academic microbiologists are offering their kit, consumables and hands in helping national testing agencies to increase their capacity. For those in isolation or in vulnerable groups, it is time for some home working and a chance to learn those new skills in bioinformatics and coding that you have really been meaning to acquire, but most importantly, write up some papers and reviews for *Microbiology*! An inevitable consequence of the pandemic was the cancellation of the annual conference of the Microbiology Society in Edinburgh, which was also a celebratory meeting for the 75th anniversary. The Society set a strong example by cancelling early, which has been fully vindicated; the news a few days later that a scientific conference organized by Biogen in Boston resulted in over 50 confirmed cases was proof, if any was needed, that this was the right decision.

For the journal, one of the most interesting events at Annual Conference would have been the official launch of Actinobase – the first *Microbiology*-sponsored community resource (https://actinobase.org/). The community of actinobacterial researchers is very strong in the UK, with a major branch of its scientific family tree descending from Professor Sir David Hopwood, a former President of the Microbiology Society, who was one of the key founders of *Streptomyces* research. David has published 32 times in *Microbiology*, from his first article in 1957 to a reflection on 40 years of studying this fascinating genus in 1999 [1]. When I was working at the Molecular Microbiology department at the John Innes Centre (JIC) (@JohnInnesCentre) in the late 1990s, David was a legendary figure who, although retired, seemed to be enjoying himself more than ever as his team at the JIC, working with Julian Parkhill and colleagues at the Sanger Institute in Cambridge, pieced together the first genome sequence of a streptomycete, namely *Streptomyces coelicolor* A3(2) [2].

Actinobase (@ActinoBase) is a Wiki-based resource for delivering a diverse range of practical knowledge about these bacteria and was conceived around the idea of transferring and updating the ‘bible’ for *Streptomyces* researchers, namely the JIC-produced *Practical Streptomyces Genetics*. However, it is much more than this, providing a broad range of information on the bacteria themselves and methods, media, plasmids, genomes and more. Importantly, it was created and is edited by a community of early-career microbiologists, some of whom are scientific great-grandchildren of Hopwood, with others from across the UK and the world. Under the guidance of senior researchers Matt Hutchings (@MattHutchings10), Kate Duncan (@kate_duncan), Morgan Feeney (@Mostlymicrobia) and Lorena Fernández-Martínez (@lore_fermar), the team are led by Sam Prudence (@Sam_Prudence) at University of East Anglia, with Tom Maclean (@TomMcLean05), Alicia Russell at the JIC (@Alisia_Russell), Craig Allen at Swansea (@Craig_allan90), Linamaría Pintor Escobar (@LinaPintorE) at Edge Hill University and the team at the University of Strathclyde of Emily Addington (@emyaddington), David Mark (@DavidRcoMark) and Laia Castaño Espriu (@Laia_Castano), with many other editors at these institutions and beyond. The editors work together with periodic edit-a-thons to add new content, developing their own skills, learning more science and making strong networks in the process. Jealous? Let us know if you want us to consider supporting your own community research portal for your favourite group of microbes.

The first paper to be highlighted in this month’s musings is appropriately about a streptomycete and has a biotechnological focus, but not around natural product production. In this paper from the group of Akihiro Iida and colleagues at Mitsubishi Life Science, Ibaraki, Japan, the authors screened their in-house collection of over 450 bacterial strains for elastase activity – an enzyme with a range of useful properties, most important in meat tenderizing. They found that the highest activity was produced by cultures of a strain of *Streptomyces* in their collection, which they then purified and identified by N-terminal sequencing [3]. They subsequently cloned and characterized the recombinant enzyme, which they showed is from a new group within the S1 serine protease family that differs from other characterized bacterial elastases, such as the *Bacillus subtilis* alkaline elastase (subtilisin YaB), in preferring small amino acids in its cleavage region, and the authors propose that it might have useful industrial applicability.

I have always been interested in symbiotic bacteria and this month we have a number of papers increasing our knowledge of bacterial symbionts of different kinds. In our first paper we focus on the α-proteobacterium *Sinorhizobium meliloti *, which forms root nodules in leguminous plants that mature to become little cell factories for fixing nitrogen gas, ultimately to organic nitrogen, whilst at the same time being fed by the plant. A new paper from Michael F. Dunn's group in UNAM, Mexico, examined the function of polyamines in the biology of this bacterium [4]. These are small polycations that are accumulated by bacterial cells for a variety of cellular functions, and which his group, working with my colleague Jane Thomas-Oates at York (@ChemistryatYork), had shown in 2018 were required for normal cell growth [5]. *S. meliloti* synthesizes a number of polyamines, including putrescine, spermidine and homospermidine, which are derived from basic amino acids such as l-lysine using ornithine decarboxylase (ODC) enzymes. In this study they inactivate the major ODC-encoding gene, *odc2*, which significantly alters the polyamines produced and results in a range of phenotypes related to exopolysaccharide (EPS) biosynthesis, oxidative stress survival, motility and biofilm formation. Given all these phenotypes, it is not surprising that there is also an effect on nodulation. The number of nodules was not reduced, but their function was poor, as reflected by decreases in plant weight and height. The authors think this nodule phenotype most likely results from the defects in EPS production, which likely underlie many of the other phenotypes, but the mechanism of this control is not known at present [4].

Another feature of some rhizobia, including the organism highlighted in the next study, *Rhizobium leguminosarum *, is their multipart genomes, often containing very large plasmids. The particularly large ones, typically over 0.5 Mb, often contain ‘core’ genes not normally found on plasmids and have been usefully called chromids to distinguish them from large plasmids; a discovery made by my York colleague Peter Young that only seems to have partly caught on [6]. The smaller plasmids in these species are usually conjugative and hence can mobilize genes between strains. They can also recombine with the larger plasmids and still conjugate, resulting in the movement of much larger pieces of DNA [7]. In this paper from Michael Hynes’s group in Calgary, Canada, the authors establish the function of genes in the *tra–trb* clusters from one of the smallest plasmids, pRleVF39b, uncovering the functions of a number of genes [8]. Many of these have similar functions in other conjugative clusters, but they discover a new gene, *trcF*, which is important for conjugation efficiency. The protein is similar to histidinol phosphatases and its precise function is still to be elucidated, although the authors were able to show that it does not play a role in regulation of the other conjugation genes.

The nitrogen-fixing bacteria are textbook examples of symbiotic bacteria living in association with plants, and a similarly well-studied symbiotic system with an animal is that of the bacterium *Vibrio fischeri* and its partner the Hawaiian bobtail squid. Here the bacteria grow in a compartment created by the squid and when the bacteria reach a certain cell density they fluoresce. This weak light shines down below the squid so that it makes less of a shadow when hunting, a process called counterillumination. The production of the fluorescent molecule is density-dependent and was a key experimental system used to elucidate the process of quorum sensing in bacteria. Although a few year old now, Bonnie Bassler telling this story is a must-see TED talk (How bacteria ‘talk’). A new paper from the group of William Soto and colleagues at the College of William and Mary, Williamsburg, Virginia, USA examines how adaptations to external pH in the free-living state of the bacterium can alter its symbiotic function [9]. Using experimental evolution, the group evolved strains with increased fitness to both acid and alkaline stress in the free-living state. Interestingly, they found that the acid-adapted lines were better able to colonize the squid and produce bioluminescence, while the alkaline ones were worse off. They conclude that this is a clear example of where selection for a free-living trait can have consequences for the efficiency of the symbiotic portion of the life cycle, which supports a similar paper published on this more generally last year, also in *Microbiology* [10].

Linking from this paper on the regulation of gene expression by changes in pH, we consider another on the same topic, but in a commensal yeast rather than a bacterium. The organism, *Malassezia furfur*, is one I have come across from working with Unilever on our body odour bacteria project, as they are also interested in scalp disorders such as dandruff, which are linked to colonization by this yeast [11]. As these yeast live on the skin surface, which contains abundant secreted lipids, they have become lipophilic, meaning that they have a complete reliance on the host for free fatty acids needed for lipid biosynthesis. To access this free pool of lipids they need to secrete lipase enyzmes to break down host lipids such as triacylglycerols. In this study from Sittinan Chanarat and colleagues from Mahidol University, Bangkok, Thailand, the authors examine the effect of external pH on the growth of the yeast [12]. They find that the yeast is fairly tolerant to a wide range of external pH values, but that levels of secreted lipases are significantly different across the pH range, being much more active at higher pH. Changes in skin pH can be caused by a wide range of factors, including inflammatory skin diseases, so the robust growth of the yeast across a wide pH range suggests that it is well adapted to these changes, while the specific induction of lipases by alkaline pH needs to be investigated further, as it suggests changes in the yeast phenotype that could result from host inflammatory responses.

The Editor’s choice for the March issue has been selected by senior editor Jörg Stülke (@JorgStulke) and is about the function of the SufT protein, required for iron sulfur cluster biosynthesis, in the bacterium *Mycobacterium smegmatis* [13]. You can read his comments on this paper on the Microbe Blog on the Society website. Finally, our latest Microbe Profile is for the bacterium *Campylobacter jejuni *, a notorious cause of gastroenteritis in humans [14]. The profile, written by Ozan Gundogdu and Brendan Wren at the London School of Tropical Medicine, UK, captures the key information about this pathogen and includes a summary of the important virulence factors that make this bird commensal bacterium a human pathogen. Acute *Campylobacter* infections are unusual in that they can have long-term sequalae, as the antibodies raised to the *C. jejuni* sialic acid-containing cell surface glycans cross-react with similar epitopes on neuronal cell glycans; this leads to the autoimmune condition Guillain–Barré syndrome in some patients. One unusual feature of ‘*Campy*’ biology is that it was the first bacterium in which a general *N*-linked protein glycosylation system was discovered by Wren and colleagues [15], and from the open questions the authors raise there are clearly still lots of interesting biological questions that need to be understood about this food-borne pathogen.

There will be lots more next month, including more news about our *Microbiology-*sponsored community resources.
